# Abdominal ultrasound performance assessment: a comparison of generic and extended OSCE rating scales

**DOI:** 10.1186/s12909-026-08994-2

**Published:** 2026-03-14

**Authors:** Simon Schneider, Daniel Stricker, Christoph Berendonk, Joanna Baron-Stefaniak, Robin Walter

**Affiliations:** 1 Institute of Primary Health Care, Faculty of Medicine, University of Bern, Mittelstrasse 43, Bern, 3012 Switzerland; 2 Institute for Medical Education, Faculty of Medicine, University of Bern, Bern, Switzerland; 3 Department of Anaesthesiology and Intensive Care Medicine, Barmherzige Schwestern Krankenhaus, Vienna, Austria

**Keywords:** Ultrasound education, Clinical evaluation, Assessment techniques, Rating scales, Near-peer teaching, Near-peer assessment

## Abstract

**Background:**

Objective Structured Clinical Examinations (OSCE) are a widely used tool for assessing ultrasound competence, yet the optimal format for its rating scales remains debated. Extended, task-specific rating scales may offer detailed guidance, while more generic scales offer broader applicability and ease of use. This study compares whether and how ratings differed when using an extended versus a generic rating scale for assessing performance in basic abdominal ultrasound skills.

**Methods:**

In this single-centre cohort study, 80 medical students participated in an OSCE for abdominal ultrasound rated by both an extended and generic rating scale in parallel. Five domains were evaluated: image settings, transducer handling, examination technique, image explanation and overall performance. Each OSCE station was rated by two assessors simultaneously, one using each scale.

**Results:**

The generic rating scale demonstrated significantly higher internal consistency (mean Cronbach’s α generic 0.803 vs. extended 0.699; *p* = 0.011) and a higher generalisability (Phi generic 0.529 vs. extended 0.466). Absolute ratings on the generic rating scale were significantly lower (performance P generic 75.38% vs. extended 77.99%, *p* < 0.001), though in-between stations difficulty was only significantly different for two stations.

**Conclusion:**

In total, the generic rating scale showed a more stringent rating with a higher internal consistency and a higher variance due to participants’ performance.

Therefore, a well-designed generic rating scale can reliably and efficiently assess basic abdominal ultrasound performance, possibly offering greater generalisability and simpler implementation than extended task specific rating scales.

**Supplementary Information:**

The online version contains supplementary material available at 10.1186/s12909-026-08994-2.

## Background

Objective structured clinical examinations (OSCE) are a common examination format for ultrasound courses as it captures its manifold facets best [1–5]. Their organisation, administration, and execution demand substantial knowledge, experience, and planning [6, 7]. The creation of the OSCE rating scale is a key component of this process [7]. OSCE assessment requires rating scales to capture as much information as possible within limited observation time. While generic rating scales have shown advantages for less technical skills [8–10], it remains debated whether this also applies to technical skills such as ultrasound [11, 12].

Accordingly, recent literature on ultrasound exams generally distinguishes into two options: task-specific (i.e. extended) rating scales [3, 13] and generic rating scales [1, 5, 14]. Both types of rating scales, generic and extended, seem to provide advantages [15]. Supporters of extended rating scales argue they offer greater reliability and validity [3, 13, 16]. In contrast, supporters of a generic rating scale contend that the use of elaborate and procedure-specific tools may not always provide a better estimate of performance than scales relying on general competencies [5, 15, 17, 18]. Furthermore, generic rating scales are easier to develop and can be quickly adapted to new OSCE stations, thereby simplifying OSCE organisation and coordination. Despite those facts, there has never been a head-to-head comparison of a generic and a task-specific extended rating scale for abdominal ultrasound; they have all been proposed and validated individually.

In this study, we aimed to compare a generic and an extended rating scale evaluating the same domains. We investigated whether and how ratings differed in terms of internal consistency, generalisability and performance scores when using an extended versus a generic rating scale for assessing performance in basic abdominal ultrasound skills.

## Methods

### Context

The Young Sonographers, a subsection of the Swiss Society for Ultrasound in Medicine (SGUM/SSUM), have provided courses on basic abdominal ultrasound since 2019, comprising five hours of e-learning and 16 h of practical training taught by near-peer tutors [19, 20]. Following a revision of this course on abdominal ultrasound [21], existing OSCE stations and their task-specific extended rating scales [13] were updated based on rater feedback, a thorough literature review and a final expert discussion (unpublished thesis, available on request). Based on this revision, two rating scales were created using identical rating domains: one extended, task-specific version with very precise instructions and one generic scale providing only very general prompts.

### Study design

This single-centre cohort study was conducted at the University of Bern to compare both rating scales in the abdominal ultrasound OSCE. Eighty medical students (3rd − 6th year; 65% female; mean age 23 years) who had completed the course and consented to participation were included. 64 participants reported less than five hours of ultrasound experience prior to the course, 14 reported more than five hours and two participants did not provide a response. The study aimed to examine how both rating scales (extended, task-specific vs. generic) performed in terms of internal consistency and generalisability. Performance scores P (expressed as percentages out of a maximum of 30 points) were analysed across the rating scales, series and stations to better understand the sources of variability.

### OSCE organisation

Twelve OSCE stations each assessed one course’s learning objective through a 4-minute practical ultrasound task. Stations were combined into eight six-station series (A1–D2; Appendix 1). Participants alternated between examiner and model roles.

Each series was completed by two groups (*n* = 11/12) of participants, except for Series D1 and D2, which were completed by only one group each (*n* = 6).

### Rating scales

**Fig. 1:**
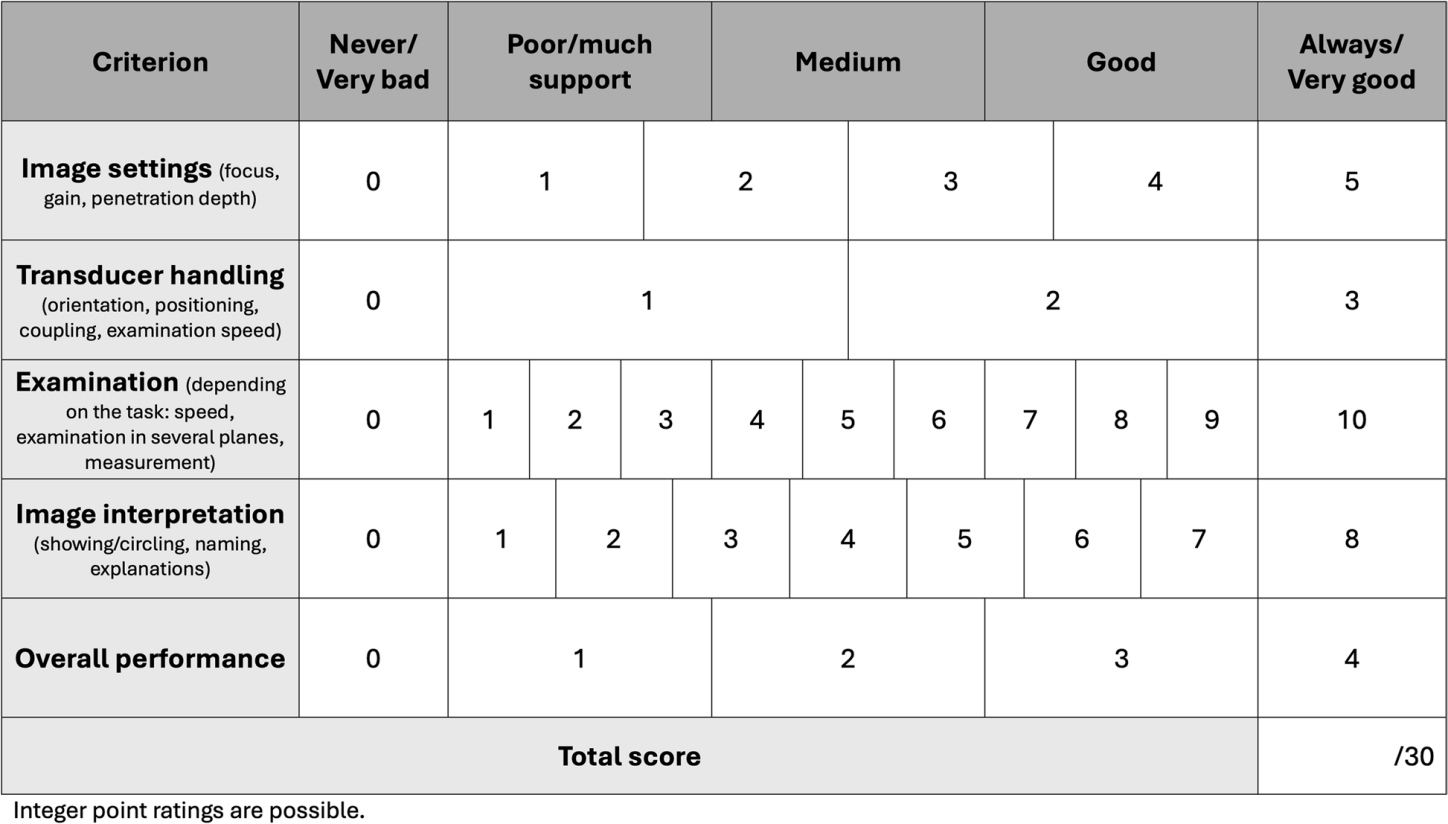
Generic rating scale. Integer point ratings are possible

Both rating scales used identical domains - image settings, transducer handling, examination technique, image interpretation, and overall performance - with a total of 30 points. The extended scale contained task-specific descriptors (Appendix 2), whereas the generic scale provided brief, uniform prompts (Fig. 1).

### Data collection

Each OSCE station was rated by two assessors simultaneously, one using each scale. Assessors were randomly assigned and included two SGUM-accredited physicians and 23 trained near-peer tutors with ≥ 1 year of teaching experience. Separate information sessions were held for each group, which conveyed basics for the OSCE itself and the study format, as well as instructions on how to use the rating scales, followed by two exemplary training videos to ensure a common frame of reference [22]. For each video existed an optimal rating score proposed by experienced ultrasound assessors, which was used to lay ground for the discussion among the raters.

### Statistical analysis

To evaluate reliability, internal consistency was quantified using Cronbach’s α, as a number between 0 and 1 [23]. Internal consistency using Cronbach’s α could be inadvertently inflated if a rater distributes the same number of points to a same item for any reason, a bias known as straight-lining. It is therefore inevitable to provide additional measures for reliability, such as Generalisability Theory (GT). GT was used to assess the influence of non-rating scale-related factors on the final score (i.e. how much of variance was explained by the participants performance). As the OSCE was designed for criterion-referenced interpretation of absolute performance rather than norm-referenced ranking, only absolute generalisability coefficients (Phi) were calculated. Phi coefficients were calculated, ranging from 0 to 1, with 1 meaning that 100% of the variance in scores is due to the respondent’s performance [24]. The Phi coefficients were calculated for the following model: “(examinee : series) x (station : series)”. Examinees were nested within series, and stations were treated as a random facet within each series. The model did therefore assume that not all participants or series encountered all stations. For summative decisions, a Cronbach’s α of 0.8 [3, 12] and a Phi coefficient of 0.65 is usually deemed adequate [25]. Although our exam is summative, it is not high-stakes, as failing this exam does not affect students’ regular progress in their studies. Therefore, we considered a Cronbach’s α of 0.7 and a Phi-coefficient of 0.5 as adequate for this OSCE.

A multivariate split-plot ANOVA was used to examine how rating scale (within-subjects), series and OSCE station (between-subjects) contributed to variability in performance scores (α ≤ 0.05). Effect sizes were reported using partial η^2^, interpreted as small (< 0.07), medium (0.07–0.14) and strong (> 0.14). The level of significance was adapted using a post-hoc Bonferroni correction. If relevant differences between rating scales were found, paired-sample t-tests were performed to identify responsible OSCE stations.

All statistical analyses were performed using SPSS for Windows, version 28 (IBM, Armonk, NY, USA). For the Generalizability Theory (GT), G_String, version 2019 was used.

### Ethical considerations

The ethics committee of Bern, Switzerland declared this study was exempt from the Human Research Act and the need for a comprehensive ethical appraisal (BASEC number Req-2023-00254). Collected data was treated after being anonymised. All participants were informed about the study, then provided informed consent to participate or could withdraw without consequences on them receiving the course certificate. This study was conducted in accordance with the ethical principles outlined in the Declaration of Helsinki.

## Results

### Internal consistency and generalisability of rating scales

Our results indicate that internal consistency was higher for the generic scale than for the extended rating scale (Cronbach’s α 0.803 vs. 0.699; *p* = 0.011, Table 1). Across stations, Cronbach’s α ranged from 0.50 to 0.83 for the extended and 0.74–0.87 for the generic scale (Appendix 4). The generalisability analysis (GT) revealed a Phi coefficient of 0.466 for the extended and 0.529 for the generic scale (Table 1), suggesting that generic scales show greater performance-related variance than extended scales. The main results for both rating scales are indicated in Table 1. In a subsequent decision study, reliability increased steadily as more stations were added, with the extended rating scale reaching a projected Phi ≥ 0.65 at 13 stations and the generic rating scale reaching the same target at ten stations (see Appendix 5).

**Table 1:** Participants’ performance P, Cronbach’s, and Generalisability Phi per rating scale

		Extended rating scale	Generic rating scale	Significance
Cronbach’s α	Mean	0.699	0.803	*p* = 0.011*
	*Min.*	*0.500*	*0.739*	
	*Max.*	*0.827*	*0.873*	
Generalisability (Phi)		0.466	0.529	
Performance P [%]	Mean	77.99	75.38	*p* < 0.001*
	*SD*	*7.13*	*7.67*	

*Indicated *p*-values derive from ANOVA or t-test analyses between rating scales, all significant

### Influence of rating scale on examinees’ performance

In multivariate testing, using a split-plot ANOVA we found a significant interaction between type of rating scale and performance P (F(1, 461) = 22.95, *p* < 0.001, η² = 0.047; small effect), in other words the two rating scales rated participants’ performance significantly different with the generic rating scale attributing lower scores than the extended scale.

Additionally, “series” and “station” showed significant effects (F(7, 461) = 2.22, *p* = 0.032, η² = 0.033; F(11, 461) = 3.95, *p* < 0.001, η² = 0.086; small–medium effects) on participants’ performance P. Though, a significant interaction between type of rating scale and station was found (F(11, 461) = 2.782, *p* = 0.002, η^2^ = 0.062, small effect size), the interaction between type of rating scales and series was not significant (*p* = 0.108), suggesting the difference between scales was balanced across the 12 series.

Finally, we tested individual OSCE stations between rating scales to identify whether all stations are responsible for the significant overall difference using paired samples t-tests. Analyses yielded statistically significant differences between participants’ performance P only for the stations “retroperitoneum” and “hepatic veins” (*p* < 0.001, Appendix 3).

## Discussion

In this single-centre cohort study, we evaluated whether a generic and an extended rating scale for abdominal ultrasound skills yield comparable ratings on examinees’ performance in OSCE. We found that a generic rating scale rated participants’ performance with a higher internal consistency and generalisability, while attributing lower rating scores overall.

Our findings are aligned with broader medical education literature questioning the superiority of task-specific rating scales in performance-based exams. Early on, Regehr et al. showed that global rating scales demonstrate higher reliability and validity than checklists [10], mirroring our observation that the generic scale produced more consistent ratings. Studies comparing generic domain-based versus extended checklist-based OSCE assessments likewise report that generic ratings better discriminate between levels of competence [8, 9]. Although some authors argue that generic ratings scales may be less appropriate for technical skills [11, 12], our results show that generic rating scales can also yield reliable assessments in a highly technical domain such as ultrasound.

Within ultrasound education, our results complement the ongoing debate on the usefulness of detailed, task-specific rating scales. Hofer et al. advocated for extended scales to capture procedural complexity [3]. However, in our study, extended scales produced inflated scores particularly in anatomically complex stations (hepatic veins, retroperitoneum), suggesting that extensive rating scales may inadvertently mask performance differences. More recent work supports moving away from highly granular rating scales: Ma et al. identified critical point-of-care ultrasound rating items but recommended restricting assessment to essential behaviours [15], and Tolsgaard et al. emphasised evaluation of core domains, such as image optimisation, systematic examination, and interpretation, rather than long lists of procedural steps [17]. Our direct comparison provides empirical support for this shift, indicating that generic scales may better capture ultrasound performance by focusing on broader domains that reflect the integrated nature of the skill.

Taken together, our findings suggest that generic rating scales may be a suitable choice when designing OSCE assessments for basic abdominal ultrasound. Even though our findings suggest using generic rating scales for summative OSCE decisions, more detailed feedback on performance - guided by precise, extensive rating scales - might be supportive in formative OSCE, despite lower psychometric efficiency. Further research should explore whether these findings extend to less technical areas of medical education and whether faculty and near-peer assessors perform differently when using generic scales. The effect of rater experience also remains unexplored by our study, as generic rating scales may require greater rater expertise because they provide fewer task-specific cues and depend more on holistic judgement. However, their simplicity seems to also benefit near-peer raters in time-constrained OSCEs when combined with frame-of-reference training, as used here.

### Strength and limitations

This study is the first to directly compare two rating scale formats for abdominal ultrasound OSCE assessment. To minimise cross-contamination of rating styles between assessors, we separated examiners into different break rooms and rotated them regularly. Our use of a diverse examiner group, including faculty and trained near-peer tutors, reflects real-world practice and supports generalisability for technical skills examination such as ultrasound. However, because only a small proportion of examiners were faculty, subgroup analysis was not feasible; results might differ in an assessor cohort composed entirely of faculty. Transferability to other OSCE contexts may also be limited by the narrow and well-defined task of abdominal ultrasound, where assessors share a relatively uniform mental model of optimal performance; conditions that may not hold in broader clinical domains. Additionally, the transferability of the results could be limited due to the study’s single-centre cohort design.

Although generalisability theory accounts for measurement error arising from design facets, it does not explicitly model response-process artefacts, though it is much less receptive to straight-lining than Cronbach’s α alone. Since in our study both reliability measures agree in their general statements and since our ANOVA showed significant differences between scales and stations, it is unlikely that straight-lining strongly influenced our results.

## Conclusion

For abdominal ultrasound OSCEs, generic rating scales appear to offer more stringent rating behaviour, higher internal consistency, and greater performance-related variance than extended scales. A well-designed generic rating scale can reliably and efficiently assess basic abdominal ultrasound competence, potentially offering greater generalisability and simpler implementation than extended, task-specific rating scales.

## Supplementary Information

Supplementary Material 1.

## Data Availability

The used data are available from the corresponding author upon request.

## Abbreviations

OSCE: Objective structured clinical examinations
SGUM/SSUM: Swiss society for ultrasound in medicine
ANOVA: Analysis of variance
GT: Generalizability theory
