# Supplementary material for: Abdominal ultrasound performance assessment: a comparison of generic and extended OSCE rating scales
Source: BMC Med Educ. 2026 Mar 14;26:653. doi: 10.1186/s12909-026-08994-2 (PMC13101218; doi:10.1186/s12909-026-08994-2)
Supplement: Supplementary file 1 — Supplementary Material 1. [file 12909_2026_8994_MOESM1_ESM.docx]

| **Appendix 1** The 8 different series | | | | | | |
| --- | --- | --- | --- | --- | --- | --- |
|  | **Station 1** | **Station 2** | **Station 3** | **Station 4** | **Station 5** | **Station 6** |
| Serie A1 | Thyroid | Aorta | Pancreas | Right LL | Hepatic veins | Left kidney |
| Serie A2 | Spleen | Right kidney | Bladder | Left LL | Vena portae | Gallbladder |
| Serie B1 | Aorta | Right LL | Spleen | Bladder | Left kidney | Hepatic veins |
| Serie B2 | Pancreas | Vena portae | Left LL | Thyroid | Gallbladder | Right kidney |
| Serie C1 | Vena portae | Left kidney | Aorta | Pancreas | Thyroid | Left LL |
| Serie C2 | Right LL | Bladder | Right kidney | Spleen | Hepatic veins | Gallbladder |
| Serie D1 | Thyroid | Gallbladder | Pancreas | Hepatic veins | Right LL | Right kidney |
| Serie D2 | Spleen | Left LL | Bladder | Left kidney | Vena portae | Aorta |
| *LL = liver lobe* | | | | | | |

**Appendix 2** Example - Extended Rating Scale

| **OSCE** | Name participant: |
| --- | --- |
| Date: | Name assessor: |

| Station 8 (Gall bladder) | | | | | | | | | | | | | | | | | |
| --- | --- | --- | --- | --- | --- | --- | --- | --- | --- | --- | --- | --- | --- | --- | --- | --- | --- |
| A patient presents with subcostal tenderness in the right medio clavicular line. 1) Please examine the entire gallbladder. 2) Measure the wall thickness. 3) Show an ultrasound artefact caused by the gallbladder and explain its origin.  *Subcostal oblique and right MCL longitudinal., close examination & wall measurement, explaining and showing an artefact of the gallbladder.* | | | | | | | | | | | | | | | | | |
| **Image settings** | | | | | | | | | | | | | | | | | |
| **Are the settings made adequately throughout the examination:**  Adequately and independently sets the appropriate magnification, focus and gain during the entire examination | | | | | | | | | | | | | | | | | |
| 5 - always | 4 | | | 3 - partly | | | 2 | | | 1 – with lots of support | | 0 - never | | **0 - 5** | | |  |
| **Transducer handling during the entire examination** | | | | | | | | | | | | | | | | | |
| **Is the transducer adequately guided during the examination:**  Correct orientation of the transducer, positioning at the correct location, sufficient coupling, appropriate pressure, adequate movements, adequate examination speed | | | | | | | | | | | | | | | | | |
| 3 - always | | | 2 - mostly | | | 1 - rare | | | 0 - never | | | | **0 - 3** | | |  | |
| **Examination technique** | | | | | | | | | | | | | | | | | |
| **Examination of the organ:** | | | | | | | | | | | | | | | | | |
| ● Completely examinated and in two levels, even and adequate tempo  ● Completely examinated and in two levels, tempo inadequate  ● not complete in two levels or one level complete  ● Only one level poorly or with help  ● Nothing seen at all; no image can be held despite help  *integer point ratings are possible* | | | | | | | | | | | | | | | **8**  **6**  **4**  **2**  **0** |  | |
| **Measurement of the wall thickness:** | | | | | | | | | | | | | | | | | |
| ● Correct, perpendicular to the wall on the liver side  ● Measurement only with manual help  ● measured incorrectly despite manual help  *integer point ratings are possible* | | | | | | | | | | | | | | | **4**  **2**  **0** |  | |
| **Image explanation** | | | | | | | | | | | | | | | | | |
| ● Shows and explains an artefact correctly (e.g. shadow artefact, distal sound enhancement, slice thickness artefact)  ● Shows an artefact with no or incorrect explanation  ● Shows and explains no artefact  *integer point ratings are possible* | | | | | | | | | | | | | | | **6**  **3**  **0** |  | |
| **Overall performance** | | | | | | | | | | | | | | | | | |
| 4 – very good | | 3 - good | | | 2 - sufficient | | | 1 - insufficient | | | 0 - clearly insufficient | | **0 - 4** | | |  | |
| **Total score (max. 30)** | | | | | | | | | | | | | | |  |  | |

| **Appendix 3** Paired Samples Test | | | | |
| --- | --- | --- | --- | --- |
| **Checklist** | | **Mean** | **Std. Deviation** | **Significance - Two-Sided p** |
| Thyroid | P extended - P generic | 2.08333 | 14.70871 | 0.376 |
| Retroperitoneum | P extended - P generic | 6.33333 | 11.24196 | <.001 |
| Pancreas | P extended - P generic | 4.41667 | 14.38952 | 0.059 |
| Right lobe of the liver | P extended - P generic | 3.50000 | 11.98170 | 0.072 |
| Left lobe of the liver | P extended - P generic | -2.25000 | 11.77635 | 0.234 |
| Hepatic vein star | P extended - P generic | 9.33333 | 10.62706 | <.001 |
| Vena portae | P extended - P generic | 4.33333 | 14.64100 | 0.069 |
| Gall bladder | P extended - P generic | -2.25000 | 9.73692 | 0.152 |
| Right kidney | P extended - P generic | -.16667 | 11.24577 | 0.926 |
| Left kidney | P extended - P generic | -.08333 | 13.78482 | 0.970 |
| Spleen longitudinal | P extended - P generic | 2.83333 | 10.44726 | 0.094 |
| Bladder | P extended - P generic | 3.16667 | 15.37656 | 0.200 |
|  | | | | |

| **Appendix 4** Cronbach’s Alpha and Difficulty P per Station | | | |
| --- | --- | --- | --- |
| **Station** | **Typ of rating scale** | **Cronbach’s Alpha** | **Difficulty P** |
| Thyroid | extended | 0.688 | 83.9 |
|  | generic | 0.812 | 80.8 |
| Retroperitoneum | extended | 0.811 | 80.2 |
|  | generic | 0.844 | 75.9 |
| Pancreas | extended | 0.823 | 80.4 |
|  | generic | 0.831 | 75.3 |
| Right lobe of the liver | extended | 0.805 | 81.3 |
|  | generic | 0.873 | 75.8 |
| Left lobe of the liver | extended | 0.5 | 73.6 |
|  | generic | 0.82 | 74.9 |
| Hepatic vein star | extended | 0.784 | 84.2 |
|  | generic | 0.772 | 76.3 |
| Vena portae | extended | 0.776 | 75.8 |
|  | generic | 0.818 | 71.4 |
| Gall bladder | extended | 0.615 | 80.3 |
|  | generic | 0.833 | 82.8 |
| Right kidney | extended | 0.549 | 74.9 |
|  | generic | 0.752 | 74.6 |
| Left kidney | extended | 0.827 | 73.8 |
|  | generic | 0.739 | 73.1 |
| Spleen longitudinal | extended | 0.655 | 75.6 |
|  | generic | 0.776 | 74.3 |
| Bladder | extended | 0.553 | 81.6 |
|  | generic | 0.765 | 78.7 |

**Appendix 5** GT Decision study

| Type | Generalisability model | n stations | Phi |
| --- | --- | --- | --- |
| extended | (examinee : serie) x station | 4 | 0.368 |
| generic |  | 4 | 0.428 |
| extended |  | 5 | 0.421 |
| generic |  | 5 | 0.484 |
| extended |  | 6 | 0.466 |
| generic |  | 6 | 0.529 |
| extended |  | 7 | 0.504 |
| generic |  | 7 | 0.567 |
| extended |  | 8 | 0.538 |
| generic |  | 8 | 0.600 |
| extended |  | 9 | 0.567 |
| generic |  | 9 | 0.628 |
| extended |  | 10 | 0.592 |
| generic |  | 10 | 0.652 |
| extended |  | 11 | 0.615 |
| generic |  | 11 | 0.673 |
| extended |  | 12 | 0.636 |
| generic |  | 12 | 0.692 |
| extended |  | 13 | 0.654 |
| generic |  | 13 | 0.709 |
| extended |  | 14 | 0.671 |
| generic |  | 14 | 0.724 |
| extended |  | 15 | 0.686 |
| generic |  | 15 | 0.738 |
| extended |  | 16 | 0.699 |
| generic |  | 16 | 0.750 |
